# Designing a National Rapid Vaccine Coverage Survey in low-resource settings: Experiences from the Democratic Republic of the Congo 2018–2023

**DOI:** 10.1016/j.vaccine.2025.126956

**Published:** 2025-04-19

**Authors:** Eric M. Mafuta, Aimée M. Lulebo, Jean-Bosco N. Kasonga, Nono M. Mvuama, Christophe L. Luhata, Nicole A. Hoff, Dalau M. Nkamba, Sydney Merritt, John Samuel Otomba, Branly K. Mbunga, Aimé M.W.B. Cikomola, Anne W. Rimoin, Jean-Crispin Mukendi, Jean Bernard LeGargasson, Cyril Nogier, Léon Kinuani, Marcellin Mengouo Nimpa, Daniel K. Ishoso, Adèle N. Mudipanu, Deo Manirakiza, Didine K. Kaba, Jean K. Nyandwe, M. Carolina Danovaro-Holliday, Amine El Mourid, Paul-Samson D. Lusamba

**Affiliations:** a Kinshasa School of Public Health, University of Kinshasa, Kinshasa, Democratic Republic of the Congo; b Expanded Program for Vaccination, Minister of Public Health, Hygiene and Prevention, Kinshasa, Democratic Republic of the Congo; c University of California Los Angeles, Department of Epidemiology, CA, Los Angeles, USA; d World Health Organization, DRC EPI Office, Kinshasa, Democratic Republic of the Congo; e Gavi, The Vaccine Alliance, Geneva, Switzerland; f UNICEF DRC Child Survival Office, Kinshasa, Democratic Republic of the Congo; g Immunization, Vaccines and Biologicals Department, World Health Organization, Geneva, Switzerland; h Bill and Melinda Gates Foundation, London, United Kingdom

**Keywords:** Vaccination coverage, Survey methodology, Democratic Republic of the Congo, Routine immunization, Health system strengthening

## Abstract

In the Democratic Republic of the Congo (DRC), estimating vaccine coverage (VC) has traditionally relied on large-scale surveys such as the Demographic and Health Surveys (DHS) and the Multiple Indicator Cluster Surveys (MICS). However, these surveys are infrequent, costly, and lack the granularity needed for decision-making at the health district or zone (HZ) level. This paper describes the development by the Kinshasa School of Public Health (KSPH), and technical partners of a Vaccine Coverage Survey (KSPH VCS), adapted from the World Health Organization (WHO) guidelines, which aims to provides timely, cost-effective, and representative estimates of VC at the HZ level.

The KSPH VCS adopted a cross-sectional design and a multi-stage sampling approach to sample households at the HZ level. It uses Health Area as cluster in spite of Enumeration Area, and extends the eligibility age range from 12 -23 months to 6–23 months. The sample size for each HZ was calculated using vaccine coverage provided in MICS-2018. It integrates assessments of barriers and enablers to vaccination. Since 2023, it has included malaria indicators. Since its inception in 2018, it has expanded nationwide, covering all 26 provinces of the DRC by 2022.

Findings from the KSPH VCS provides estimates at the HZ level that could be combined to provincial and national estimates. Results have been instrumental in evaluating national immunization strategies, including the Mashako Plan and informing Presidential Forums on immunization. They have informed resource allocation, operational planning, and policy decisions at both national and provincial levels as they provided granularity needed for operational decision-making at the HZ level. Its results have also contributed to global immunization estimates, including the WHO/UNICEF Estimates of National Immunization Coverage (WUENIC).

The KSPH VCS demonstrates the feasibility of a locally led, cost-effective, and adaptable VC survey in a low-resource setting. Its success highlights the potential for similar methodologies to be implemented in other low- and middle-income countries seeking to improve immunization monitoring and health system performance.

## Highlights

• A context-adapted survey approach (KSPH VCS) has been developed and executed. in DRC.
• The KSPH VCS is a timely, cost-effective approach for estimating vaccine coverage.
• KSPH VCS is implemented annually with results informing routine immunization policy.

## 1 Background

Vaccination is a highly cost-effective and successful public health intervention that contributes to reducing global child morbidity and mortality due to vaccine-preventable diseases (VPDs) [[1], [2], [3]]. Since the 1974 establishment of the Expanded Program Immunization (EPI), routine immunization (RI) systems have been implemented worldwide to ensure every child, and woman (neonatal tetanus-toxoid vaccination), is fully vaccinated against VPDs. The EPI strategy uses RI coverage as a major indicator of program performance to target resources and identify gaps in coverage.

The Demographic and Health Surveys (DHS) and Multiple Indicator Cluster Surveys (MICS) are multimillion-dollar nationally representative surveys conducted every 3–5 years by a host of international and national partners [[4], [5], [6], [7]]. These surveys are often broadly focused with internationally validated questionnaires and based on a representative sample of clusters at the sub-national level. However, their sampling strategy allows them to estimate indicators at the first subnational level (provinces) but rarely at the second (health zones, equivalent to health districts); vaccination coverage modules are included within a larger framework. While the DHS and MICS are implemented using standard international metrics, they often only provide sub-national coverage estimates with low statistical precision. Depending on implementation processes and country-specific needs, adding questions to these modules is often difficult and limited; the set methodology helps to ensure surveys are standardized, which may, conversely may not allow for the collection of sufficient data to be validated as official coverage results for a country [8]. Furthermore, unlike vaccination-specific surveys, DHS and MICS do not include qualitative questions regarding vaccination barriers and enablers, or reasons for no vaccination – with only rare exceptions such as the Nigeria MICS/NICS 2017 and 2021 [9]. DHS and MICS result dissemination often occurs months or years following completion, rendering these data of limited use for informing immediate operational-level changes [5,8].

Beyond larger surveys, many low- and middle-income countries (LMICs) have relied on administrative coverage data to estimate vaccination coverage [10]. In DRC for instance, administrative immunization data is collected at individual health facilities and includes records of immunization sessions as well as monthly Health Area (HA)-level vaccination reports. These estimates are aggregated at the Health Zone (HZ) level and integrated into the District Health Information System 2 (DHIS2) database. While Data Quality Audits (DQA) or Data Quality Self-Assessments (DQS) have been proposed methods for evaluating the quality of this administrative data [11], comparisons with population-based surveys have shown gross overestimates of coverage by administrative data sources in some countries [12]. For example, when pairing administrative and survey vaccination estimates from 71 countries, the median differences in reported vaccination for all antigens ranged from 26 to 30 % with administrative estimates consistently higher than the survey-based estimate [12,13]. World Health Organization (WHO) and UNICEF triangulate administrative coverage data with survey estimates, and in many cases, surveys set the coverage level in the WHO/UNICEF Estimates of National Immunization Coverage (WUENIC) [14,15].

Historically, obtaining vaccine coverage data in the DRC has been challenging, with information on immunization services exclusively collected as part of the DHS or MICS or through smaller regionally specific subnational surveys [16]. Additionally, during the 2018 DRC MICS, the vaccine coverage estimates were considered inconsistent and not used to inform WUENIC. Beyond these larger surveys, the WHO regularly updates guidance on conducting cluster Vaccine Coverage Surveys (VCS) for estimating vaccine coverage at national and subnational levels, with the last update in 2018 (next revision is expected for 2025–2026) [17]. Originally, the EPI survey methodology included the selection of 30 clusters in each estimated area and seven eligible households with children 12–23 months per cluster [18]. However, the 2018 update recommends probability sampling aligned with current standard methodology in major household surveys, visiting health facilities to identify for vaccine records of children without vaccination cards, and adapting the survey methodology to country-specific contexts, which may include variable routine immunization (RI) schedule recommendations [17]. As of 2024, the recommended RI schedule for children in DRC includes the following vaccines: bacillus Calmette-Guérin (BCG); polio (oral and inactivated vaccines); diphtheria, tetanus toxoid, pertussis-containing vaccine (DTP-containing vaccine) with hepatitis B and *Haemophilus influenzae* type b (pentavalent vaccine); conjugate pneumococcus vaccine; rotavirus; yellow fever, and measles-containing vaccines [19,20]. Children are considered “fully immunized” after they have received all recommended RI doses before their first anniversary or by 11 months. Nevertheless, VCS can be just as costly as the DHS and MICS, if subnational precision is desired, and typically rely on external consultants hired by the funder or technical partners, which can increase costs and limit their ability to enhance local capabilities or capacity. As the second largest African country by area, and third by population, with varied resource availability, there are numerous challenges to conducting VCS in the DRC [21].

In the DRC, administrative estimates of vaccine coverage have ranged from 80 % to over 200 % for individual antigens and districts, while data from nationally conducted surveys have not shown real improvements in vaccine coverage over the past decade, with a decline in DTP3 from 60.5 % in the DHS 2013–14 to 47.6 % in the MICS 2018, though both surveys are not directly comparable [22,23]. In addition, since 2017, the DRC has continued to experience the emergence of circulating vaccine-derived poliovirus type 1 and 2 cases, as well as yellow fever and recurrent measles outbreaks. Driven in part by the results of the MICS and ongoing outbreaks of VPDs, the DRC EPI and its partners developed an emergency plan to reinvigorate the RI system, called the Mashako Plan. The Mashako Plan was launched in October 2018 and aimed to increase full immunization coverage by 15 percentage points from the MICS 2018 results in nine initial provinces (Kinshasa, Haut Katanga, Kwilu, Kasai, Ituri, Mongala, Tshuapa, Tanganyika, and Haut Lomami), within 18 months [20]. The Mashako Plan targeted specific challenges in vaccine availability, delivery, and accessibility at all levels of the RI system and incorporated routine monitoring and evaluation [20]. As a part of the routine evaluation, a feasible, cost-effective, independent and timely evaluation method was needed to ensure that the plan was delivering measurable improvements in immunization at the national and subnational scale.

When implementing coverage surveys, challenges, in LMIC settings or other low-resource areas, include knowing and updating the sampling frame, ensuring distributed site selection, and ensuring the probability sampling method for implementation [10]. Key criteria for development of a coverage assessment methodology in the DRC included costs, feasibility of regular and comparable data collection, ease of implementation, adaptability of the sampling strategy to include all HZs, and external validity of results produced. To assess the progress made towards improving full immunization coverage and to understand the spatial variation in vaccination coverage in the context of varying vaccine delivery strategies and the implementation of the Mashako Plan, the Kinshasa School of Public Health (KSPH) at the University of Kinshasa developed and implemented a yearly independent VCS adapted from the 2018 WHO guidelines. The major objective of this strategy was to generate coverage estimates at the HZ level which could be compiled for provincial-level estimates of immunization coverage, alongside qualitative information related to vaccination barriers and enablers.

## 2 Development and implementation of the KSPH VCS

In 2019, Acasus and KSPH collaborated to develop a VCS methodology specific to DRC. Administratively, the DRC's health system operates on three levels: central, intermediate, and peripheral. The central level sets policies and regulations, and provides tertiary care. It includes the Ministry of Health, the General Secretary with central directorates, specialized programs, national hospitals, and the General health inspectorate. The intermediate level functions at the provincial level, responsible for translating policies into actionable strategies and providing technical supervision. It includes the Provincial Minister in charge of Health, a Provincial Health Division and Inspectorate, and provincial hospitals. The peripheral or the operational level, or Health Zones (HZ), implements primary health care services, including immunization and basic health care. The DRC has 519 HZs. Each HZ is established as a homogenous region based on geographical, demographic, socio-cultural and economic criteria. Each HZ is further divided into Health Areas (HAs), each served by a Health Center (HC) catering to 5000 to 10,000 people. This structure reflects the DRC's decentralized health management, where primary health care services in theory can reach even the most remote areas.

**Fig. 1:**
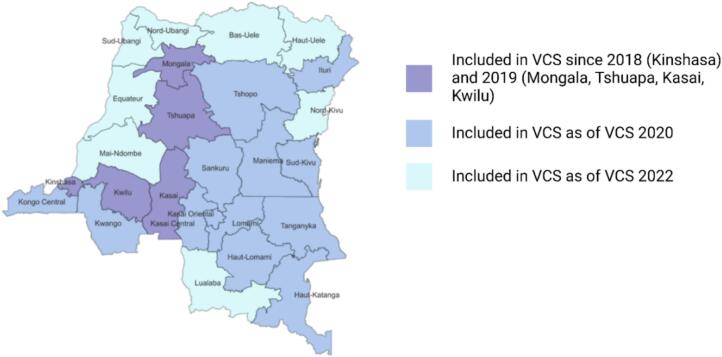
Provinces included in the KSPH VCS by first year of implementation (2018-present).

The KSPH VCS adopts a cross-sectional design based on the WHO methodology but modified to ensure representativeness at the HZ level, as opposed to just provincial level representativeness. Representativeness at the HZ level is important as the HZ is the operational unit of the DRC health systems in terms of resource allocation, health care planning and implementation. Prior to national implementation, the VCS approach was first piloted in the Kinshasa province in September 2018. Between 2019 and 2023, KSPH continued to expand and refine the study methodology with the support of WHO, UNICEF, EPI, and funding partners, leading to the development of the KSPH WHO-adapted methodology (KSPH VCS). Following the initial deployment, the number of provinces included in the VCS increased each year until 2022, when all 26 provinces were included **(**Fig. 1**)**.

The KSPH VCS methodology differs in several key respects from previous studies mainly DHS or MICS **(**Table 1**)**. The KSPH VCS approach extended the age eligibility range from 12-23 months to 6–23 months, enabling the timely availability of data for children who are still in basic vaccination schedule, which included measles and yellow fever doses at 9 months. The sample size for health zones within a province was determined using provincial vaccine coverage estimates from MICS-2018, with an accepted error margin of 10 % and a design effect of 1.5 (Table 2). The KSPH VCS also included estimates of how many children nation-wide had never received a vaccine—results were presented separately for 6–11-month and 12–23-month groups to ensure comparability with other surveys. As of the KSPH VCS conducted in 2023, a malaria indicator module has also been included as part of the administered questionnaire. These malaria indicators include not only questions related to the disease burden but also the use of bed nets for children under five years old living in eligible households and as of the most recent iteration of the VCS, have included a smaller sub-sample of rapid diagnostic testing to better understand the parasitic prevalence among under five years old children nationwide. Additionally, the KSPH VCS conducted in 2024 was also expanded to include questions regarding neonatal tetanus vaccination and intermittent preventive treatment for women who had given birth in the last 12 months.

**Table 1:** Key Adaptations of the KSPH VCS Methodology.

**Survey Component**	**WHO Standard Surveys**	**KSPH Adapted Method (KSPH-VCS)**
**Eligible Age Range**	12–23 months⁎	6–23 months
**Sampling**		
Sampling Scheme	2-stage/3-stage	3-Stage
Strata	Admin1 (province-equivalent)	Health Zone (district-equivalent)
Number of Clusters	Variable by strata	5+ per PSU
Primary Sampling Unit (PSU)	Enumeration Area (EA)	Health Area (HA)
Secondary Sampling Unit (SSU)	Household	Segmentation of the HA
Tertiary Sampling Unit (TSU)	N/A	Household
**Method to Ascertain Vaccination Status**	Vaccination Cards, Caregiver Recall, Health Facility Registries	Vaccination Cards, Caregiver Recall, Health Facility Registries

⁎ Usually 12–23 months, but guidance includes flexibility and most countries use 12–23 months and 24–35 months, the latter mainly where vaccines are also recommneded in the second year of life.

**Table 2:** Effective sample size calculation for each province using MICS 2018 data.

**N°**	**Province**	**deff**	**Z**	**p (%)**	**q = (100-p) (%)**	**d (%)**	**Min Sample ZS**
1	Haut - Katanga	1.5	1.96	45.5	54.5	10	142.9
2	Haut - Lomami	1.5	1.96	35.7	64.3	10	132.3
3	Kasaï Central	1.5	1.96	41.3	58.7	10	139.7
4	Kasaï Oriental	1.5	1.96	27.9	72.1	10	115.9
5	Kasaï	1.5	1.96	13.9	86.1	10	69.0
6	Kinshasa	1.5	1.96	41.9	58.1	10	140.3
7	Kongo Central	1.5	1.96	48.0	52.0	10	143.8
8	Kwango	1.5	1.96	16.3	83.7	10	78.6
9	Kwilu	1.5	1.96	14.3	85.7	10	70.6
10	Lomami	1.5	1.96	26.9	73.1	10	113.3
11	Ituri	1.5	1.96	40.0	60.0	10	138.3
12	Maniema	1.5	1.96	6.6	93.4	10	35.5
13	Mongala	1.5	1.96	8.2	91.8	10	43.4
14	Sankuru	1.5	1.96	2.6	97.4	10	14.6
15	Sud Kivu	1.5	1.96	48.9	51.1	10	144.0
16	Tanganyika	1.5	1.96	21.2	78.8	10	96.3
17	Tshopo	1.5	1.96	20.7	79.3	10	94.6
18	Lualaba	1.5	1.96	21.1	78.9	10	95.9
19	Maindombe	1.5	1,96	11.9	88.1	10	60.4
20	Bas-Uele	1.5	1.96	23.8	76.2	10	104.5
21	Haut-Uele	1.5	1.96	17.5	82.5	10	83.2
22	Nord-Kivu	1.5	1.96	79.2	20.8	10	94.9
23	Sud-Ubangi	1.5	1.96	26.9	73.1	10	113.3
24	Nord-Ubangi	1.5	1.96	16.6	83.4	10	79.8
25	Equateur	1.5	1.96	28.5	71.5	10	117.4
26	Tshuapa	1.5	1.96	15.4	84.6	10	75.1

The KSPH VCS Interviewees included the head of household and guardians (mother, father, other relatives) of all eligible children in a household. In the absence of home-based records (HBRs, also known as vaccination cards) to verify vaccination status, the survey methodology relied on guardian recall or declaration. As of 2020, if a child lacked an HBR to verify their vaccination status, the interviewing team would attempt to identify their vaccination record at the health facility where the respondent reported the child was last vaccinated. Photographs of HBRs and facility-based records are taken and used to verify data, mainly related to vaccination dates, as recommended for VCS [17], and GPS coordinates are provided for both households and health facilities. Finally, the KSPH VCS was able to nest serosurveys since the 2020 VCS for two or three provinces [[24], [25], [26]]. As this is not the main feature of the methodology, this aspect is not further discussed here.

**Fig. 2:**
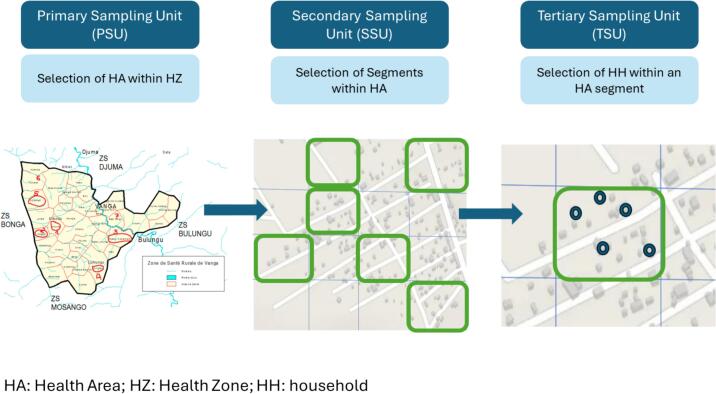
Sampling Strategy of the KSPH VCS Approach.

The KSPH VCS uses multi-stage sampling to reach eligible households **(**Fig. 2**)**. Previous VCS studies were conducted either only in a section of selected HZs or in clusters randomly selected at the provincial level, which did not guarantee representation of every HZ within the sampling frame [27]. By including clusters at the health area-level (several health areas make a health zone) within HZs, the KSPH VCS produces representative HZ estimates, that are combined to produce estimates at the provincial and national levels with high statistical precision; it also facilitates doing small area estimation using geospatial techniques [28]. KSPH VCS sampling is based on health system organization, and cluster definition is independent of census population estimates. All provinces and HZs are considered as strata in the data collection [29]. In addition, as the HZ is the operational unit of the RI system, estimates at this level can be directly linked to programmatic action.

HZs are divided into HAs [30], and the HA was chosen as the primary sampling unit (PSU) instead of the enumeration area of the census system (EA). An HA (Health Area) is the operational unit of a Health Zone (HZ) for health care delivery. Information collected at the HA level provides insights into how health care services function. All HAs are enumerated and regularly updated in DHIS2 (Table 3). In contrast, an EA (Enumeration Area) is an administrative unit created for census purposes. In the DRC, EAs are linked to the administrative system rather than the health system. Although an EA is generally smaller than an HA, its list is not regularly updated, as a national census has not been conducted in DRC since 1984. Therefore, in the first stage, five HAs and two potential replacement HAs were randomly sampled in each HZ at the national coordination level. The DHIS2 database contains a complete, annually updated list of HAs [31], which were then arranged in alphabetical order and numbered for each health zone; HAs were then selected using a random number generator application with replacement protocol. If a duplicate HA was drawn, the sampling process was repeated. Replacement HAs were included for use only in insecure regions for team safety. For HZs with fewer than 5 HAs, all HAs were selected and the sample size in each cluster was increased **(**Fig. 2**)**.

**Table 3:** Characteristics of the DRC Health zones and provinces.

Variables	n	%
Health area		
Mean number of health area	18	
Median number of health area	18	
Minimum number of health area	4	
Maximum number of health area	41	
Health zones with 10 health areas and less	43	8.3
Health zone with health area between 11 and 20	301	58.0
Health zone with health areas between 21 and 25	115	22.2
Health zone with health areas between 26 and 30	44	8.5
Health zone with health areas between 31 and 41	16	3.1
Total number of health areas in 2022	9470	
Health zones		
Total number of health zones	519	
Mean number of health zones in provinces	19.9	
Median number of health zones (Min, Max)	17 (11–36)	

In the second sampling stage, segments of the HAs (secondary sampling unit, SSU) are drawn at random. In 2020 and 2021, the segment consisted of streets or villages. A list of segments was drawn up by the team supervisor with the support of local political, administrative and health authorities, and 30 % of these segments were randomly selected. This sampling strategy did not guarantee the dispersion of households, as it was subject to the pocketing effect. Additionally, data collectors systematically tended to sample streets or villages near the health center or main roads, avoiding remote locations. Therefore, in 2023, the HA segmentation procedure was updated to a quadrant procedure in which 16 segments were generated within the HA, with six segments randomly drawn a priori to improve household dispersion and avoid pocket effects (Fig. 2). This segmentation was done using both digital and handwritten maps provided by local authorities.

In the third stage of sampling, in each sampled segment, eligible households (tertiary sampling unit, TSU) were systematically sampled by the survey team. To establish the sampling frame, households (HHs) were enumerated using a land-survey and an electronic form. Eligible HHs (those with children aged 6–23 months) were identified following enumeration. In 2020 and 2021, the entire segment corresponding to 30 % of streets or villages was visited by the survey team. All HHs were visited to identify eligible HHs through a simple question to the head of the HH or guided by a community health workers, and 30 eligible HHs were sampled among all identified eligible HHs. In 2022 and in 2023, survey team progressively visited all HHs in each of the six sampled segment to identify respectively 18 eligible HHs and 15. Five eligible HHs were then systematically sampled among them totalizing 30 HHs for the HA. In each eligible HH sampled, data were collected for all eligible children through CAPI (computer-assisted personal interviewing) using an electronic survey tool hosted in Survey CTO and GPS coordinates were collected.

Data collection was conducted by a survey team with health science backgrounds composed of five people with at least two women. Data collection took approximately one workday per HA to the survey team and one day and half taking into account malaria data collection. This time was higher in 2020/2021 as survey team had to enumerate a wider segment of the HA. HHs identified were revisited three times if people were absent. The absent HH was replaced by the following in the sampling roster. By province, there were at least three to eight survey teams all under one provincial supervisor. Provincial supervisors were trained during a seven-day workshop including a pretest and a debriefing by the coordination team in Kinshasa. Survey teams in each province were trained by the provincial supervisor supported by a coordination team member including pretest and debriefing in each provincial capital. Each team surveys 3–4 HZs. The survey protocol and preliminary results are discussed and validated by a steering committee of EPI program officers and technical partners.

The KSPH VCS is submitted for ethical review annually; participants provided written informed consent between 2018 and 2020 and have provided oral informed consent since 2021. While personal identifiers are collected to triangulate home-based and facility-based data, they are kept confidential, and de-identified during analysis. Data are stored on cloud-based servers protected by password. All surveyors and data users signed a non-disclosure data agreement. Data quality is ensured by the appropriate recruitment of supervisors and surveyors, training, timely internet monitoring and daily data checks.

Following data collection, analysis and estimate generation were completed by a KSPH team in Kinshasa using the Vaccination Coverage Quality Indicator (VCQI) specifications [32]. Analysis by the KSPH team was completed using Stata Statistical Software (College Station, TX). Annually, data from the KSPH VCS survey is disseminated through meetings with the national EPI team, provincial health authorities as well as external partners. Beyond presentation, and an annual report is also produced by the KSPH team for online publication.

Beyond the implementation of a VCS sampling methodology, which built on DHS and MICS survey implementation in DRC, the KSPH VCS was also designed to be conducted annually, independent of EPI. To ensure this, the cost and timeliness of survey result availability were key design parameters. A sustainable survey approach would need to be feasibly funded by the DRC itself and results disseminated within months of completion to have functional data points to inform actionable health policy decisions.

## 3 Results

### 3.1 Data Generated

**Fig. 3:**
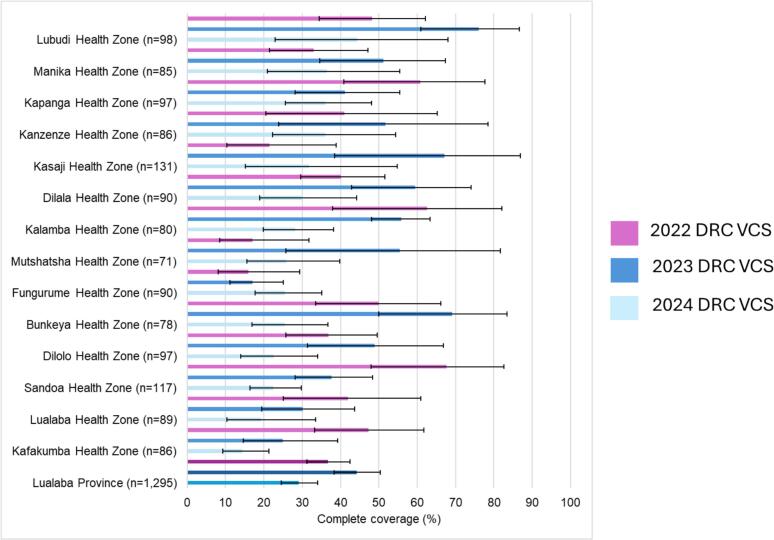
Health Zone Level Estimates of Fully Vaccinated in Lualaba Province, VCS 2024, 2023 and 2022. Fully vaccinated defined as having received BCG, three doses of Pentavalent, three doses of Pneumococcal conjugate vaccine, three doses of Rotavirus vaccine, three doses of oral polio vaccine, Measles vaccine, and yellow fever vaccine. (For interpretation of the references to colour in this figure legend, the reader is referred to the web version of this article.)

The KSPH VCS surveys were conducted in September 2018 (Kinshasa only), October 2019 (4 additional provinces), October 2020 (18 provinces), January 2022, February 2023 and March 2024 (26 provinces) for a total of five surveys (Table 4). These surveys were able to estimate coverage (for all vaccines, both individually and combined) for every HZ and every province across the country. Vaccine coverage estimates are presented along with their confidence intervals to illustrate the range of possible values, accounting for variations between clusters, specifically HAs. Variations in survey estimates reflect changes in immunization service performance at the (HZ level, while wider confidence intervals indicate differences in immunization service provision among HAs. For example, in Lualaba province, data could be shared individually per HZ and aggregated at the provincial level (Fig. 3).

**Table 4:** Point Estimates of Vaccination Coverage of selected vaccines by VCS Year.

**Year**	**Number of Provinces**	**Number of HZ**	**Number of HA**	**Number of Children 12–23 months**	**Card Possession**	**Zero dose**⁎	**Pentavalent-1**	**Pentavalent-3**	**Measles**	**Fully vaccinated**⁎⁎
**2018–19**	**5**	The 2018–2019 KSPH VCS results are not reported as no report was published with all five provinces as a cohesive VCS. Every province was assessed separately.								
**2020**	**18**	374	1870	46,093	33.5	16.8	83.2	67.6	68.5	52.5
**2021–22**	**26**	511	2460	51,054	53.5	19.1	80.9	60.3	55.9	41.5
**2023**	**26**	503	2523	47,880	62.7	19.3	80.7	61.1	56.1	45.3
**2024**	**26**	509	2712	46,990	59.4	19.5	80.5	57.6	52.2	38.3

⁎ ”Zero-dose” here is defined as not having received any dose of a pentavalent (DTP-HepB-Hib) vaccine as per the Immunization Agenda 2030 Monitoring and Evaluation (M&E) framework and Gavi 5.0 [1,2].

⁎⁎ Fully vaccinated defined as having received BCG, three doses of Pentavalent, three doses of Pneumococcal conjugate vaccine, three doses of Rotavirus vaccine, three doses of oral polio vaccine, Measles vaccine, and yellow fever vaccine.

Beyond primary data collection of vaccine coverage at the HZ level, the KSPH VCS also generates a comprehensive dataset which includes reasons for non-vaccination, and more recently has used the behavioral and social drivers of vaccination (BeSD) framework [33]. Additionally, data collection is monitored real-time if internet access is available, with daily data quality checks for team GPS distribution, the percentage of persons who report having received a HBR and the number of children for who facility-based data is needed. HBR pictures or the facility-based records, if available, are uploaded with each individual survey.

The VCS data and results were regularly shared and presented at the national level and at the provincial level. The provincial health division and authorities were also informed of the results via WhatsApp channels, official provincial reports, publication on the KSPH website and provincial reviews. The data was available on an easy-to-access platform or dashboard for provincial level and in report or excel format for district level. The report that is shared with immunization partners includes multiple analyses done at the HZ level. Secondary analysis with the KSPH VCS data has been included in publications highlighting zero-dose and under-vaccination rates in DRC [34,35], and evaluations of vaccination initiatives and policies such as the Memorandum of Understanding in Haut Lomami, Tanganyika and Lualaba provinces [36]. Other secondary analyses are underway, including one on timeliness and drop-out, one related to the quality of home-based records and one exploring area of residence vs. area of vaccination.

### 3.2 Programmatic impact

KSPH VCS results have been presented to and validated by the EPI Research Steering Committee. The coverage results are used to evaluate the immunization program and the Mashako plan, and during the presidential forums [20]. At the global level, the results from the 2021, 2022 and 2023 surveys were incorporated into the calculation of WUENIC country indicators [37].

Furthermore, the results from the KSPH VCS are now widely used by the EPI program, the Ministry of Health, and external partners (Bill and Melinda Gates Foundation, Gavi, UNICEF, USAID, WHO, etc.) in designing and evaluating immunization interventions in the country. Specifically, the EPI program relies on yearly VCS data for the operational plans for each province as well as in the semiannual reviews in which provinces document progress towards reaching vaccine coverage targets outside of the standard administrative coverage data. For example, following the sharing of results, the provincial level launched a process of reflection on the factors that had led to these results and indicated that these results made it possible to focus supervision and planning efforts on low-performing health zones.

The VCS data has been central to the bi-annual Mashako Plan reviews since 2019. In 2022, the EPI conducted a data triangulation exercise using the KSPH VCS surveys and administrative data that led to the revision of the time series of coverage estimates reported to WHO and UNICEF that ultimately informed WUENIC. The VCS data was also central to evaluation at the Presidential Forums in 2019, 2021 and 2023. The VCS results have shaped action plans of the national EPI, as well as served as the foundation of the 2022 Gavi Equity Accelerator Funding (EAF) applications and 2024 Health Systems Strengthening (HSS4) applications by the country. By providing estimates at an operational level, this adapted approach has also broadened understanding of zero-dose rates and associated factors across the DRC and informed interventions designed improve vaccine coverage. The latest results of the survey conducted in 2023 and 2024 led to a fundamental requestioning the current approaches being implemented to improve coverage and reduce the number of zero-dose children. Additionally, the VCS anonymized datasets can be requested for use in secondary data analysis for publications by partners [34,38].

### 3.3 Timeliness of VCS results

**Fig. 4:**
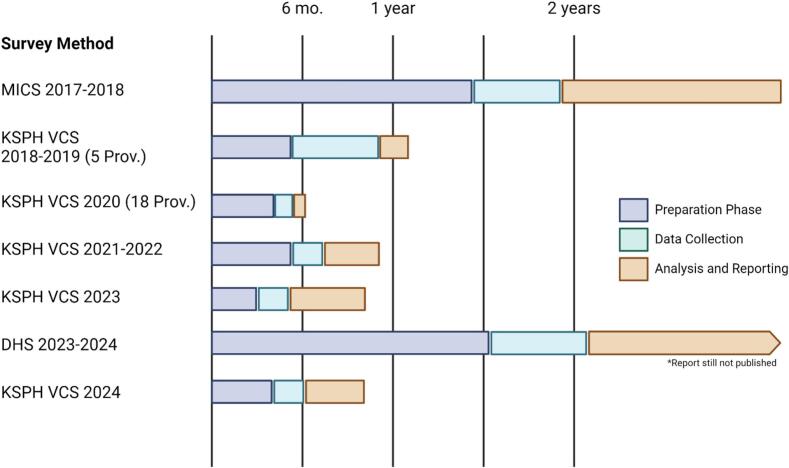
Time Estimates of DRC Vaccination Coverage Surveys.

VCS have been agile in their design, analysis and result sharing [4,39]. As the KSPH VCS has typically focused solely on vaccination data and more recently malaria indicators, planning, implementation and dissemination took on average less than 6 months from initial planning to preliminary result dissemination **(**Fig. 4**)**. This has allowed for more routine data sharing of national coverage results. With this result turnaround, the adapted approach provides actionable, relevant results which can inform operational-level policy changes and interventions.

### 3.4 Costs and in-country capacity development

The KSPH VCS presents sustainable, cost-effective approach to gathering annual vaccine coverage estimates. Costs include both personnel and operational costs. Previous estimations in Kinshasa province alone [16] cost upwards of 300,000 USD **(**Table 5**)**. Additionally, as of 2023, the VCS questionnaire has expanded to include malaria indicators - highlighting the adaptability and scalability of the approach, with a low marginal cost increase (now a total cost of 2.1 million USD).

**Table 5:** **Cost Comparisons of the KSPH VCS over time.** No costing data was available for the 2018 (Kinshasa) and 2019 (Mongala, Tshuapa, Kasai, Kwilu) implementation of the KSPH VCS.

	SCOPE	Total Cost (USD)	Cost per Province	COST PER Health Zone	Cost per Cluster
**2020 KSPH VCS**	18 Provinces	$1,000,653.89	$55,591.81	$2675.55	$535.11
**2021–22 KSPH VCS**	National	$1,652,297.67	$63,549.88	$3233.46	$671.66
**2023 KSPH VCS**	National + Malaria Indicators	$ 1,854,756.00	$ 71,336.77	$3687.39	$ 735.14
**2024 KSPH VCS**	National + Malaria Indicators, Rapid Diagnostic Testing + Neonatal tetanus	$ 2,129,029.25	$ 81,885.74	$4182.77	$ 785.04

**Associated References.**

1. Lindstrand A. IA2030 MONITORING AND EVALUATION FRAMEWORK: Annex 1. World Health Organization (WHO).

2. Gavi tVA. Phase V (2021–2025): Gavi, the Vaccine Alliance; 2024 [Available from: https://www.gavi.org/our-alliance/strategy/phase-5-2021-2025.

One major cost reduction was the reliance and integration of a local research institution, the Kinshasa School of Public Health, due to the organization's history of VCS implementation. Costs were also reduced by limiting the number of international consultants needed – no external consultants were hired for the implementation of the VCS in DRC, while strengthening local capacities and engaging people who know the country context. The KSPH team has benefited from technical assistance of WHO and VCQI manual development experts, and from partners universities and research groups through other research activities.

While designing the VCS, in-depth conversations between financial partners and implementation teams also led to the removal of extraneous costs such as car rental costs, a priori household identification and sampling costs. The DRC national VCS was intentionally designed to be a more affordable approach than other coverage surveys, both historically in DRC and other similar low-resource contexts.

## 4 Discussion

The KSPH-VCS approach provides a contextually feasible and quality approach for estimating both provincial and health zone level RI coverage data. Unlike past coverage surveys in the DRC, this approach uses multi-stage sampling from every individual health zone and includes an improved qualitative section related to drivers and barriers to immunization.

### 4.1 Programmatic Impact of the VCS

The KSPH VCS is not only a functional approach for evaluation of RI, but also for larger, more holistic, evaluations of health outcomes. Based on the success of the VCS implementation, there has been continued funding through partner consortiums. The results of these national surveys now serve as the basis for programmatic evaluation and operational interventions. As the go-to figures for immunization data following a shift from past administrative methodology, the KSPH VCS has become an important tool for public health practice.

Methodologically, this approach uses probability sampling, in which each eligible household and child had a known, non-zero probability of being selected. By avoiding census-based probability-proportional-to-size sampling cluster selection, the KSPH VCS sampling frame uses the health area as a cluster and is not subject to potential biases caused by inaccurate or out-of-date census data, particularly in the DRC where most recent census was in 1984. In a country of the size and heterogeneity of DRC, this approach is also more feasible and easier to implement. The VCS study is based on health system organization, and the definition of clusters is independent of the census population estimates.

As an approach that is entirely conducted in DRC by KSPH, the data from these surveys has been well received and trusted by the national EPI and Ministry of Health as their own data. Opposed to the past DHS or MICS, the KSPH VCS has the flexibility to include EPI and partners in the steering committee and has been designed to be a more transparent and tailored assessment to the realities of the EPI in DRC. All aspects of data collection, analysis and dissemination are available for review, and openly shared with partners. From an external, international perspective, these data are also being used for assessment—there has been results consensus from local health care workers to the DRC Ministry of Health and international organizations such as the WHO and UNICEF.

However, the KSPH VCS continues to be improved with each year of its implementation. Notably, one improvement in future implementation will be the reduction of the size of secondary clusters so that each segment contains only two or three eligible households, and that each segment is fully or exhaustively surveyed within the primary sampling unit.

### 4.2 Expansion of the Mashako Plan

Coverage surveys, such as this adaptation, can be used to track vaccination progress and are essential indicators of interventions like the Mashako Plan [20]. While previous approaches were limited to provincial-level feedback, the KSPH VCS delivers operational-level data to inform immunization strategies. This VCS has been instrumental to monitor outcomes in initial Mashako Plan 1.0 [20], and attest to the stagnation of immunization improvements following Mashako Plan 2.0—prompting conversation that the RI revitalization program needs additional support. Beyond the Mashako Plan, the VCS has also been instrumental in the bi-annual review of immunization coverage at the Presidential Forums [20], as well as larger scale international coverage estimates such as the WUENIC. The KSPH VCS has been instrumental in the estimation of national infant immunization coverage exercise in 2022 which resulted in a revision of the historical WUENIC estimates for DRC from 2009 onwards [14,15].

Unlike the larger administrative surveys including the DHS and MICS, that take 2–3 years from initial planning phase to data dissemination, VCS have been agile in their design, analysis and result sharing. [4,39]. This has allowed for more routine data sharing of national coverage results. With this result turnaround, the adapted approach provides actionable, relevant results which can inform operational-level policy changes and interventions.

Beyond providing quality, national estimates, the KSPH VCS presents sustainable, cost-effective approach to gathering annual vaccine coverage estimates compared to using externally supported surveys. Estimations in Kinshasa province alone [16] cost upwards of 300,000 USD, while the broader DHS and MICS approaches are similarly limited in their implementation by their higher costs.

Finally, neighboring countries have shown interest in the KSPH VCS with the Central African Republic having recently implemented a similar survey, with support from 10.13039/501100007217KSPH in 2024 [40].

Methodologically, successive KSPH-VCS could adhere to a longitudinal follow-up methodology as interrupted time series at the HZ level. Even though, clusters and households are randomly selected each year and the cohorts change each year in the immunization program, as most of the vaccines are given during the first year of life, it is possible to track changes in immunization status at the HZ level. In addition, analysis of 10–11-month children provide current coverage estimate at the time of sampling while the 12–23-month cohort indicates previous year.

### 4.3 Limitations of the KSPH VCS

The KSPH VCS utilizes a multi-stage sampling strategy for reaching households and health facilities for data collection. The strategy has some disadvantages when compared with simple random sampling, such as: potential estimation variability and high sample design effects caused by pocket effects, and the tendency of households near each other to share the same vaccination status.

While earlier iterations did not include maternal tetanus-toxoid containing vaccines assessments – an integral part of EPI – the 2023 KSPH VCS has since included this component.

While not intrinsic to the VCS methodology, the limited availability of HBRs in some provinces results in a large reliance on caregiver recall, known to result in biases in vaccination status ascertainment [41]. This is partially mitigated by the facility traceback looking for records of vaccination in facilities. Encouragingly, the availability of HBRs seen has increased from 33.5 % in 2020 to 59.4 % in 2023 nationwide.

Estimations in some health zones showed wider confidence intervals. The vaccine coverage is calculated from multiple clusters (e.g., different HAs), and the interpretation of its confidence interval (CI) has to account for intra-cluster correlation (ICC) and potential variability across clusters. The CI is affected by the variability within each cluster (e.g., vaccine coverage within a HA) and between clusters (differences among HA) and the presence of clustering often increases the variance, making the confidence interval wider compared to a simple random sample. A wider CI could translate the decrease of the effective sample size at the health zone level, occurring when the vaccine coverages are highly similar within the same HA or when some HAs contributed more respondents than others, In DRC context, a wider IC probably indicate that some HAs have very different proportions, associated with geographical, operational, demographic of socio-cultural factors influencing vaccination rates.

However, wider CI are acceptable at the health zone level in program practice, as vaccine coverage surveys are generally designed with a margin of error of plus or minus 5–10 %. However, at the provincial level, aggregating data from every health zone provide a more equitable distribution of the data collected. Conversely, the larger DHS or MICS surveys, which, depending on the size and number of clusters, may miss heterogeneity by not having collected data from some health zones.

The use of the VCS data is still limited at the district level. While results are regularly shared and presented at the national level and at the provincial level using multiple channels, the VCS data has yet to be fully employed at sub-national levels and, data on how the district health managers interact with the data are still missing.

## 5 Conclusions

Here, we present an accessible, functional adaptation to the WHO Vaccine Coverage Survey methodology as designed and implemented by the Kinshasa School of Public Health. When compared to broader, international surveys such as the DHS or the MICS, this KSPH VCS approach provides granular health-zone level estimates which can direct interventions at the operational level. Additionally, over the four years of implementation, this approach has expanded beyond assessments of routine immunization alone, but instead to include broader indicators of child health country wide. While no perfect assessment for RI exists, we present a viable method specifically adapted to the DRC-context.

## Ethics considerations

All procedures were performed in compliance with relevant laws and institutional guidelines and have been approved by the Kinshasa School of Public Health IRB.

## Funding

This work was supported by the 10.13039/100000865Bill and Melinda Gates Foundation [grant number INV-035951].

## Authorship

All authors attest they meet the ICMJE criteria for authorship.

## CRediT authorship contribution statement

**Eric M. Mafuta:** Writing – review & editing, Writing – original draft, Supervision, Project administration, Methodology, Investigation, Formal analysis, Data curation, Conceptualization. **Aimée M. Lulebo:** Writing – review & editing, Supervision, Project administration, Methodology, Investigation, Conceptualization. **Jean-Bosco N. Kasonga:** Writing – review & editing, Methodology, Investigation, Data curation. **Nono M. Mvuama:** Writing – review & editing, Project administration, Methodology, Investigation, Data curation, Conceptualization. **Christophe L. Luhata:** Writing – review & editing, Supervision, Project administration, Methodology, Investigation, Conceptualization. **Nicole A. Hoff:** Writing – review & editing, Writing – original draft, Supervision, Project administration, Methodology, Funding acquisition, Conceptualization. **Dalau M. Nkamba:** Writing – review & editing, Supervision, Project administration, Methodology, Investigation, Formal analysis, Data curation, Conceptualization. **Sydney Merritt:** Writing – review & editing, Writing – original draft, Visualization. **John Samuel Otomba:** Writing – review & editing, Supervision, Project administration, Methodology, Conceptualization. **Branly K. Mbunga:** Writing – review & editing, Project administration, Methodology, Investigation. **Aimé M.W.B. Cikomola:** Writing – review & editing, Supervision, Methodology, Conceptualization. **Anne W. Rimoin:** Writing – review & editing, Supervision, Resources, Funding acquisition, Conceptualization. **Jean-Crispin Mukendi:** Writing – review & editing, Supervision, Project administration, Methodology, Conceptualization. **Jean Bernard LeGargasson:** Writing – review & editing, Supervision, Methodology, Data curation, Conceptualization. **Cyril Nogier:** Writing – review & editing, Supervision, Methodology, Conceptualization. **Léon Kinuani:** Writing – review & editing, Methodology, Conceptualization. **Marcellin Mengouo Nimpa:** Writing – review & editing, Supervision, Methodology. **Daniel K. Ishoso:** Writing – review & editing, Supervision, Methodology, Conceptualization. **Adèle N. Mudipanu:** Writing – review & editing, Supervision, Project administration. **Deo Manirakiza:** Writing – review & editing, Supervision, Conceptualization. **Didine K. Kaba:** Writing – review & editing, Supervision, Project administration, Methodology, Investigation, Conceptualization. **Jean K. Nyandwe:** Writing – review & editing, Supervision, Project administration, Methodology, Conceptualization. **M. Carolina Danovaro-Holliday:** Writing – review & editing, Supervision, Methodology, Conceptualization. **Amine El Mourid:** Writing – review & editing, Writing – original draft, Supervision, Resources, Methodology, Investigation, Funding acquisition, Data curation, Conceptualization. **Paul-Samson D. Lusamba:** Writing – review & editing, Validation, Supervision, Project administration, Methodology, Formal analysis, Data curation, Conceptualization.

## Declaration of competing interest

The authors declare that they have no known competing financial interests or personal relationships that could have appeared to influence the work reported in this paper.

## Data Availability

Data will be made available on request.

## References

[1] VaB Immunization (2020).

[2] O’Brien K.L., Lemango E., Nandy R., Lindstrand A. (2024). The immunization agenda 2030: a vision of global impact, reaching all, grounded in the realities of a changing world. Vaccine.

[3] Shattock A.J., Johnson H.C., Sim S.Y., Carter A., Lambach P., Hutubessy R.C. (2024). Contribution of vaccination to improved survival and health: modelling 50 years of the expanded Programme on immunization. Lancet.

[4] (USAID) USAfID. The Demographic and Health Survey (DHS) Rockville, MD: United States Agency for International Development; [Available from: https://dhsprogram.com/Methodology/Survey-Types/DHS.cfm.

[5] UNICEF. Multiple cluster Indicator surveys (MICS): About MICS New York, NY: UNICEF; 2024 [Available from: https://mics.unicef.org/about.

[6] Khan S., Hancioglu A. (2019). Multiple Indicator cluster surveys: delivering robust data on children and women across the globe. Stud Fam Plan.

[7] Corsi D.J., Neuman M., Finlay J.E., Subramanian S. (2012). Demographic and health surveys: a profile. Int J Epidemiol.

[8] Huang Y., Danovaro-Holliday M.C. (2021). Characterization of immunization secondary analyses using demographic and health surveys (DHS) and multiple indicator cluster surveys (MICS), 2006-2018. BMC Public Health.

[9] Rhoda D.A., Wagai J.N., Beshanski-Pedersen B.R., Yusafari Y., Sequeira J., Hayford K. (2020). Combining cluster surveys to estimate vaccination coverage: experiences from Nigeria’s multiple indicator cluster survey / national immunization coverage survey (MICS/NICS), 2016-17. Vaccine.

[10] Cutts F.T., Claquin P., Danovaro-Holliday M.C., Rhoda D.A. (2016). Monitoring vaccination coverage: defining the role of surveys. Vaccine.

[11] Bloland P., MacNeil A. (2019). Defining & assessing the quality, usability, and utilization of immunization data. BMC Public Health.

[12] Dolan S.B., MacNeil A. (2017). Comparison of inflation of third dose diphtheria tetanus pertussis (DTP3) administrative coverage to other vaccine antigens. Vaccine.

[13] Lim S.S., Stein D.B., Charrow A., Murray C.J. (2008). Tracking progress towards universal childhood immunisation and the impact of global initiatives: a systematic analysis of three-dose diphtheria, tetanus, and pertussis immunisation coverage. Lancet.

[14] Burton A., Monasch R., Lautenbach B., Gacic-Dobo M., Neill M., Karimov R. (2009). WHO and UNICEF estimates of national infant immunization coverage: methods and processes. Bull World Health Organ.

[15] Danovaro-Holliday M.C., Gacic-Dobo M., Diallo M.S., Murphy P., Brown D.W. (2021). Compliance of WHO and UNICEF estimates of national immunization coverage (WUENIC) with guidelines for accurate and transparent health estimates reporting (GATHER) criteria. Gates Open Res.

[16] Mwamba G.N., Yoloyolo N., Masembe Y., Nsambu M.N., Nzuzi C., Tshekoya P. (2017). Vaccination coverage and factors influencing routine vaccination status in 12 high risk health zones in the province of Kinshasa City, Democratic Republic of Congo (DRC), 2015. Pan Afr Med J.

[17] (WHO) WHO. (2018).

[18] Henderson R.H., Sundaresan T. (1982). Cluster sampling to assess immunization coverage: a review of experience with a simplified sampling method. Bull World Health Organ.

[19] (WHO) WHO (2024). WHO Recommendations for Routine Immunzation - Summary Tables Geneva. https://www.who.int/teams/immunization-vaccines-and-biologicals/policies/who-recommendations-for-routine-immunization---summary-tables.

[20] Lame P., Milabyo A., Tangney S., Mbaka G.O., Luhata C., Le Gargasson J.-B. (2023). A successful national and multipartner approach to increase immunization coverage: the democratic republic of Congo Mashako plan 2018–2020. Global Health: Science and Practice.

[21] Group WB. Democratic Republic of the Congo: Overview 2024 [Available from: https://www.worldbank.org/en/country/drc/overview.

[22] (2014). Enquête Démographique et de Santé en République Démocratique du Congo 2013–2014.

[23] Plan INdlSdMd. (2019).

[24] (2022). Toolkit for integrated Serosurveillance of communicable diseases in the Americas.

[25] Hayford K., Mutembo S., Carcelen A., Matakala H.K., Munachoonga P., Winter A. (2019). Measles and rubella serosurvey identifies rubella immunity gap in young adults of childbearing age in Zambia: the added value of nesting a serological survey within a post-campaign coverage evaluation survey. Vaccine.

[26] Holroyd T.A., Schiaffino F., Chang R.H., Wanyiri J.W., Saldanha I.J., Gross M. (2022). Diagnostic accuracy of dried blood spots for serology of vaccine-preventable diseases: a systematic review. Expert Rev Vaccines.

[27] Burnett E., Wannemuehler K., Ngoie Mwamba G., Yolande M., Guylain K., Muriel N.N. (2017). Individually linked household and health facility vaccination survey in 12 at-risk districts in Kinshasa Province, Democratic Republic of Congo: methods and metadata. J Infect Dis.

[28] Ferreira L.Z., Blumenberg C., Utazi C.E., Nilsen K., Hartwig F.P., Tatem A.J. (2020). Geospatial estimation of reproductive, maternal, newborn and child health indicators: a systematic review of methodological aspects of studies based on household surveys. Int J Health Geogr.

[29] Publique MdlS. PLAN NATIONAL DE DEVELOPPEMENT SANITAIRE 2016–2020 : vers la couverture sanitaire universelle Kinshasa2016. p. 97.

[30] Congo GotDRot (2001).

[31] (2025). Sanitaire SNdI. District Health Information Software 2: Democratic Republic of the Congo.

[32] (2017). (WHO) BGCWHO. User Guide for Vaccination Coverage Quality Indicators (VCQI).

[33] Organization WH (2022).

[34] Mbunga B.K., Liu P.Y., Bangelesa F., Mafuta E., Dalau N.M., Egbende L. (2024). Zero-dose childhood vaccination status in rural Democratic Republic of Congo: quantifying the relative impact of geographic accessibility and attitudes toward vaccination. Vaccines.

[35] Ishoso D.K., Mafuta E., Danovaro-Holliday M.C., Ngandu C., Menning L., Cikomola A.M. (2023). Reasons for being “zero-dose and under-vaccinated” among children aged 12-23 months in the Democratic Republic of the Congo. Vaccines (Basel).

[36] Banza Mpiongo P., Kibanza J., Kambol Yav F., Nyombo D., Mwepu L., Basame D. (2023). Strengthening immunization programs through innovative sub-national public-private partnerships in selected provinces in the Democratic Republic of the Congo. Vaccine.

[37] WHO U (2021).

[38] Ishoso D.K., Danovaro-Holliday M.C., Cikomola A.M., Lungayo C.L., Mukendi J.C., Mwamba D. (2023). “zero dose” children in the Democratic Republic of the Congo: how many and who are they?. Vaccines (Basel).

[39] Danovaro-Holliday M.C., Dansereau E., Rhoda D.A., Brown D.W., Cutts F.T., Gacic-Dobo M. (2018). Collecting and using reliable vaccination coverage survey estimates: summary and recommendations from the “meeting to share lessons learnt from the roll-out of the updated WHO vaccination coverage cluster survey reference manual and to set an operational research agenda around vaccination coverage surveys”, Geneva, 18–21 April 2017. Vaccine.

[40] (KSPH) KSoPH. (2024).

[41] Dansereau E., Brown D., Stashko L., Danovaro-Holliday M.C. (2019). A systematic review of the agreement of recall, home-based records, facility records, BCG scar, and serology for ascertaining vaccination status in low and middle-income countries. Gates Open Res.

